# Safety and diagnostic efficacy of gadoteridol for magnetic resonance imaging of the brain and spine in children 2 years of age and younger

**DOI:** 10.1007/s00247-021-05069-w

**Published:** 2021-05-05

**Authors:** Chetan C. Shah, Maria Vittoria Spampinato, Hemant A. Parmar, Osama A. Raslan, Paolo Tomà, Doris D. M. Lin, Josef Vymazal, Cesare Colosimo, David S. Enterline

**Affiliations:** 1 Pediatric Radiology, Wolfson Children’s Hospital, Nemours Children’s Health System, 800 Prudential Drive, Jacksonville, FL 32207 USA; 2 Medical University of South Carolina, Charleston, SC USA; 3 C.S. Mott Children’s Hospital, University of Michigan, Ann Arbor, MI USA; 4 Davis Medical Center, University of California, Sacramento, CA USA; 5 Bambino Gesù Children’s Hospital, Rome, Italy; 6 Johns Hopkins University School of Medicine, Baltimore, MD USA; 7 Department of Radiology, Na Homolce Hospital, Prague, Czech Republic; 8 Insitute of Radiology, Radiodiagnostica e Neuroradiologia, Fondazione Policlinico Universitario “A. Gemelli,” Universita Cattolica del Sacro Cuore, Rome, Italy; 9 Duke University School of Medicine, Durham, NC USA

**Keywords:** Central nervous system, Children, Contrast, Diagnostic efficacy, Gadolinium-based contrast agent, Gadoteridol, Magnetic resonance imaging, Safety

## Abstract

**Background:**

Neonates and young children require efficacious magnetic resonance imaging (MRI) examinations but are potentially more susceptible to the short- and long-term adverse effects of gadolinium-based contrast agents due to the immaturity of their body functions.

**Objective:**

To evaluate the acute safety and diagnostic efficacy of gadoteridol (ProHance) for contrast-enhanced MRI of the central nervous system (CNS) in children ≤2 years of age.

**Materials and methods:**

One hundred twenty-five children ≤2 years old (including 57 children <6 months old) who underwent contrast-enhanced MRI of the CNS with gadoteridol at 0.1 mmol/kg body weight were retrospectively enrolled at five imaging centers. Safety data were assessed for acute/subacute adverse events in the 48 h following gadoteridol administration and, when available, vital signs, electrocardiogram (ECG) and clinical laboratory values obtained from blood samples taken from 48 h before until 48 h following the MRI exam. The efficacy of gadoteridol-enhanced MRI compared to unenhanced MRI for disease diagnosis was evaluated prospectively by three blinded, unaffiliated readers.

**Results:**

Thirteen changes of laboratory values (11 mild, 1 moderate, 1 unspecified) were reported as adverse events in 7 (5.6%) patients. A relationship to gadoteridol was deemed possible though doubtful for two of these adverse events in two patients (1.6%). There were no clinical adverse events, no serious adverse events and no clinically meaningful changes in vital signs or ECG recordings. Accurate differentiation of tumor from non-neoplastic disease, and exact matching of specific MRI-determined diagnoses with on-site final diagnoses, was achieved in significantly more patients by each reader following the evaluation of combined pre- and post-contrast images compared to pre-contrast images alone (84.6–88.0% vs. 70.9–76.9%; *P*≤0.006 and 67.5–79.5% vs. 47.0–66.7%; *P*≤0.011, respectively).

**Conclusion:**

Gadoteridol at 0.1 mmol/kg body weight is safe, well tolerated and effective for contrast-enhanced MRI of the CNS in children ≤2 years of age.

## Introduction

Clinical signs, symptoms or adverse outcomes related to brain gadolinium retention have not been documented following the repeated administration of any gadolinium-based contrast agent (GBCA) [1–4]. Nevertheless, concern over the potential long-term risks associated with GBCA administration has led to the increased use of macrocyclic GBCAs at many pediatric imaging centers whenever contrast-enhanced magnetic resonance imaging (MRI) is deemed essential for diagnosis [5, 6]. In Europe, the use of macrocyclic GBCAs for all extrahepatic MRI indications in both adult and pediatric patients has been mandated by the suspension of all linear GBCAs for clinical use [7].

Given that migration toward the use of macrocyclic GBCAs for pediatric MRI applications reflects the belief that these GBCAs are more stable in vivo and therefore safer for potentially more vulnerable pediatric patients who have a longer life expectancy, it follows that a macrocyclic GBCA proven to clear more rapidly from brain and body tissues resulting in lower levels of retained gadolinium might be considered the GBCA of choice for the pediatric population. This is assuming no differences in safety or efficacy relative to other available macrocyclic GBCAs. Gadoteridol (ProHance; Bracco, Milan, Italy) is a macrocyclic GBCA, recently approved by the United States Food and Drug Administration (FDA) for use in children younger than 2 years of age, including term neonates [8], with r1 relaxivity similar to the r1 relaxivity values of other macrocyclic GBCAs [9]. Gadoteridol has been shown to have similar safety and efficacy profiles to those of other macrocyclic GBCAs in adults [10, 11]. Notably, however, the rate of clearance of gadoteridol from rat brain and body tissues is more rapid than that of the macrocyclic GBCAs gadobutrol and gadoterate meglumine, resulting in significantly lower levels of retained gadolinium in the first weeks and months after administration [12–15]. It remains to be determined whether more rapid elimination and lower levels of retained gadolinium occur in humans after exposure to gadoteridol. If they do, then this might have relevance for children aged 2 years and younger whose brain and cognitive functions are still developing.

The aim of this study was to assess the safety and efficacy of gadoteridol for contrast-enhanced MRI of the central nervous system (CNS) in children aged 2 years and younger since this represents a patient population for which more rapid clearance of gadolinium might be considered particularly relevant.

## Materials and methods

Patients aged ≤2 years with suspected or known disease of the CNS who had undergone contrast-enhanced MRI with gadoteridol at one of four centers in the United States or one in Italy between August 2009 and January 2019 were retrospectively enrolled. All available safety data were assessed. Additionally, a prospectively designed blinded read of images from these patients was performed to confirm the efficacy of gadoteridol in this patient population. The study design and analysis of data were as described previously for a study performed with the high relaxivity GBCA, gadobenate dimeglumine [16]. This Health Insurance Portability and Accountability Act (HIPAA)-compliant study was conducted in accordance with the International Congress on Harmonization, Good Clinical Practice, FDA regulations, and ethical principles outlined in the Declaration of Helsinki and all applicable local regulations, and was registered at www.clinicaltrials.gov (NCT03750188). Funding for the enrollment of patients was provided by Bracco. Institutional review board (IRB) approval was obtained from each participating center. A requirement for patient informed consent for the elaboration of retrospective data was waived by the local ethics committee at each center.

### Patients

Children 2 years of age or younger were included if they had received gadoteridol at a dose of 0.1 mmol/kg body weight for known or suspected enhancing disease of the brain or spine, and had complete demographic and safety data available. Enrollment was performed consecutively at each center in strict reverse chronological order from the date of local IRB approval until the predefined sample size was attained. The efficacy of gadoteridol for visualizing enhancing disease was then assessed prospectively in fully blinded fashion for all patients included in the safety population who had pre- and post-dose T1-weighted spin echo (SE)/fast spin echo (FSE) and/or gradient recalled echo (GRE)/fast field echo (FFE) images as well as T2-weighted SE/FSE and fluid-attenuated inversion recovery (FLAIR) (if acquired) images available. If the child underwent multiple MRI examinations, only the first examination that showed an enhancing lesion was considered.

### Magnetic resonance imaging

All patient exams were conducted using commercially available MRI equipment and software packages, and all major MRI manufacturers were represented. MR imaging was performed predominantly at 1.5 tesla (T) (*n*=40, 32.0% [Signa HDxt; GE Healthcare, Milwaukee, WI; Achieva and Ingenia; Philips Healthcare, Best, the Netherlands; Magnetom Avanto and Espree; Siemens Healthineers, Erlangen, Germany]) or 3.0 T (*n*=84, 67.2% [Discovery 3 T MR750, GE Healthcare; Ingenia, Philips Healthcare; Magnetom Skyra and Verio, Siemens]). Only one patient was imaged using a 1.0-T MRI system (Panorama HFO; Philips Healthcare). Acquired images included T1-W spin echo (T1 SE), T1-W gradient echo (T1 GRE), T2-W FSE, and T2-W FLAIR acquisitions before contrast injection, and T1 SE and T1 GRE acquisitions after injection of gadoteridol at a manual bolus dose of 0.1 mmol/kg body weight (0.2 mL/kg). Due to the retrospective nature of the study, a dose of 0.1 mmol/kg ±25% by volume administered was considered acceptable for patient inclusion.

### Efficacy assessments

All images acquired at the time of the MRI examination were anonymized and transferred in standard DICOM (Digital Imaging and Communications in Medicine) file format for subsequent prospective assessment by three independent neuroradiologists (J.V., D.S.E. and C.C., each with >30 years of experience in pediatric neuroradiology) unaffiliated with the enrollment centers and fully blinded to all patient clinical profiles and imaging information. Images were evaluated using the thin client of the TeraRecon AquariusNet server (v 4.4.5.36; San Mateo, CA). The three readers performed independent assessments of pre-contrast images alone (including T1, T2 and T2 FLAIR acquisitions) and combined pre- plus post-contrast T1 images, with the images displayed in randomized order in two reading sessions separated by at least 1 month to minimize recall bias.

Assessment of lesion visualization on pre-contrast and pre- plus post-contrast images was performed in terms of three co-primary endpoints: lesion border delineation, visualization of lesion internal morphology and lesion conspicuity versus background. Assessments were performed using 4-point scales for each parameter from 1 (poor), through 2 (moderate), and 3 (good), to 4 (excellent). A score of zero was assigned by default whenever a lesion was not identified on either image set after lesion matching. Similar 4-point assessment scales have been used for image evaluation in children younger than 2 years old [17, 18]. Thereafter, patient diagnoses were made at disease (neoplastic versus non-neoplastic) level and at specific diagnosis level from a list of 54 coded diagnoses. Diagnoses made by the blinded readers were subsequently matched with the final on-site patient diagnosis made by the original investigating radiologist.

Finally, each reader was asked whether the post-contrast images provided additional information over pre-contrast images, as described elsewhere [18]. Additional information included whether enhancement revealed an abnormality not seen on the pre-contrast images; whether it improved visualization of the size, extent and/or margins of a lesion (better conspicuity); or whether the pattern of enhancement was useful in predicting the grade, histological type, vascularity, and/or aggressiveness of a lesion or documented the activity or aggressiveness of non-neoplastic processes.

### Safety assessments

The safety of gadoteridol in children younger than 2 years old was evaluated based on the information available in the clinical records of the enrolled patients. Since children underwent contrast-enhanced MRI as part of a clinical routine and were enrolled retrospectively for this analysis rather than prospectively, the safety information available reflects the safety monitoring and reporting performed for each individual child at the five investigating centers. As is the case for all contrast-enhanced MRI exams with all types of GBCA, monitoring was performed for any untoward medical occurrence during the time frame associated with the administration of gadoteridol. For the purposes of this study, any untoward medical occurrence reported in the 48 h after the administration of gadoteridol was considered an adverse event, even if the event was not causally related to the administration of gadoteridol. Safety data recorded by the investigating radiologist based on the experience/expertise of the investigational team at each site (including investigating radiologists, clinicians and anesthesiologists when applicable) included clinical adverse events and, when available, vital signs, and electrocardiogram (ECG) and clinical laboratory determinations. Standard MedDRA (Medical Dictionary for Regulatory Affairs) reporting was used, with all reported adverse events coded and summarized by system organ class and preferred term, by intensity and by causal relationship to the administration of gadoteridol. As generally defined in regulatory studies, there was a reasonable possibility (doubtful, possible, probable or definite) of a relationship to gadoteridol administration if the event followed a reasonable temporal sequence from the administration of gadoteridol and followed an established response pattern even if the event could have been caused by the patient’s clinical state, a concomitant therapy, another diagnostic/interventional procedure, or if the event could not be reasonably explained by the patient’s clinical state, a concomitant therapy or another diagnostic/interventional procedure. Similarly, events were considered to have a reasonable possibility of a relationship to gadoteridol if the report of the event contained conflicting data and/or dubious or insufficient/poor evidence. Conversely, there was no reasonable possibility of a relationship to gadoteridol administration if the event was considered definitely due to causes separate from the administration of gadoteridol. A list of adverse events possibly associated with exposure to gadoteridol is provided in the prescribing information for ProHance [8] and is comparable to lists for all GBCAs.

If an adverse event was considered by the investigating radiologist and anesthesiologist to be related to sedation or anesthesia, this was recorded as part of the event description. Individual patient-specific case report forms documented all safety data recorded by the investigating radiologist during the 48 h after the contrast-enhanced MRI examination. When laboratory data were available, the normal ranges for the parameters measured were included. The last laboratory measurement made within 48 h before administration of gadoteridol was taken as the baseline value.

### Statistical analysis

All statistical tests were performed using SAS software (version 9.3; SAS Institute, Cary, NC) and were considered significant for *P*<0.05. Continuous measurements were reported as mean±standard deviation (SD) while categorical assessments were described as number (%). The change in lesion visualization from pre-contrast images alone to combined pre- plus post-contrast images was determined for the three co-primary endpoints (lesion border delineation, visualization of lesion internal morphology and lesion conspicuity) at the patient level based on averaged scores assigned to all individual detected lesions for the same image set and patient. Comparison was performed using paired *t*-tests.

Diagnostic accuracy for the differentiation of neoplastic from non-neoplastic disease and for correct lesion diagnosis at specific disease level was determined separately for pre-contrast and combined pre- and post-contrast images for each reader. The difference in diagnostic accuracy between pre-contrast and combined pre- and post-contrast image sets was assessed using McNemar’s test. The final diagnosis made by the investigating radiologist based on all available clinical, surgical and diagnostic information was considered the reference standard for diagnostic performance determinations.

Inter-reader agreement together with 95% confidence intervals (CI) for the differentiation of neoplastic from non-neoplastic disease and for correct lesion diagnosis at specific disease level was assessed using generalized weighted kappa (κ) statistics. Agreement was classified as excellent (κ values >0.8), good (κ=0.61–0.8), moderate (κ=0.41–0.6), fair (κ=0.21–0.4) or poor (κ≤0.2) [19].

A sample size of 120 patients was calculated to provide >85% power based on a McNemar’s test of equality in accuracy, assuming an expected difference in accuracy between pre-contrast and combined pre- and post-contrast image sets of 10% with 15% discordant pairs.

## Results

Overall, 125 patients were enrolled across the five participating centers (30, 21, 14, 50 and 10 patients at centers 1 to 5, respectively). All 125 patients met the inclusion criteria, had pre- and post-dose efficacy data available, and were included in analyses of both safety and gadoteridol efficacy. Demographic details are shown in Table 1. All were term infants. Patient ages ranged from 1 day to 24 months, with a mean of 8.1 months. The age distribution included 39 (31.2%) between 12 and 24 months, 29 (23.2%) between 6 and <12 months, 40 (32.0%) between 1 and <6 months and 17 (13.6%) who were <1 month. The mean weight of enrolled patients was 7.6±3.2 kg (range: 2.1–15.5 kg) and the mean height was 66.2±12.5 cm (range: 45–92 cm). An MRI examination of the brain was performed in 112 (89.6%) cases and MRI of the spine was performed in 13 (10.4%) cases.

**Table 1:** Patient demographic characteristics and details regarding type of magnetic resonance (MR) examination and sedation during examination

Demographic		Overall	0 to <1 month	1 to <6 months	6 to <12 months	12 to 24 months
Number of subjects		125	17	40	29	39
Male/female		70/55	12/5	15/25	19/10	24/15
Age (months)	Mean±SD	8.1±7.0	8.5±6.0^a^	2.7±1.6	8.3±1.9	17.1±3.1
	Range	0.0–24.0	1–21	1.0–5.0	6.0–11.0	12.0–24.0
Weight (kg)	Mean±SD	7.6±3.2	3.4±0.5	5.4±1.7	8.4±1.7	11.1±1.7
	Range	2.1–15.5	2.3–4.4	2.1–9.1	5.2–11.2	8.6–15.5
Height (cm)	Mean±SD	66.2±12.5	50.8±2.3	57.5±8.6	69.7±5.9	79.5±5.6
	Range	45–92	48–55	45–91	58–79	69–92
Type of exam	Brain	112	16	37	27	32
	Spine	13	1	3	2	7
Dose (mmol/kg)	Mean±SD	0.101±0.02	0.103±0.01	0.103±0.03	0.1±0.01	0.099±0.01
Sedation/anesthesia^b^	Yes	77	4	13	24	36
	No	44	13	26	4	1
	N/A	4	0	1	1	2

*N/A* not available, *SD* standard deviation

^a^Age is reported in days rather than months

^b^Patients may have received more than one drug for sedation

Conscious sedation and/or anesthesia before the examination was reported for 77 (62%) patients without complications. Forty-four patients did not receive sedation or anesthesia before the examination. No information could be retrieved from clinical records for four subjects. Administered drugs for sedation/anesthesia included propofol in 53/77 (68.8%) cases. Other drugs, either alone or in combination with propofol, included thiopental, fentanyl, midazolam, nitrous oxide, dexmedetomidine, succinylcholine, rocuronium bromide, cisatracurium besilate, etomidate and sevoflurane.

At least one finding of relevant medical history was reported for 124 (99.2%) patients. Commonly reported medical history findings were oncological, occurring in 45 (36.0%) patients, and congenital, occurring in 26 (20.8%) patients.

Serum creatinine values obtained within 2 days before the administration of gadoteridol were available for 74 patients (Table 2). Estimated glomerular filtration rate (eGFR) values calculated using the Schwartz formula for patients <2 years [20] were reported for 60 patients with available height data. The mean and median serum creatinine and eGFR values reported for each age group were consistent with those of similarly aged children with normal age-related renal function [21]. No children experienced any events or symptoms that necessitated post-exam serum creatinine determinations.

**Table 2:** Serum creatinine values and estimated glomerular filtration rate (eGFR)

Demographic		Total	0 to <1 month	1 to <6 months	6 to <12 months	12 to 24 months
Serum creatinine (mg/mL)	Subjects	74	13	27	16	18
	Mean±SD	0.30±0.15	0.47±0.18	0.27±0.13	0.24±0.12	0.27±0.08
	Range	0.10–0.70	0.16–0.70	0.10–0.70	0.10–0.50	0.10–0.44
eGFR (mL/min/1.73m^2^)	Subjects	60	11	22	13	14
	Mean±SD	124.2±64.1	59.4±35.0	118.8±55.6	140.4±66.6	168.4±51.3
	Range	31.5–306.8	31.5–154.7	40.5–265.5	65.3–306.8	106.3–300.7

*SD* standard deviation

### Efficacy results

Lesions were detected by the three readers in 108 (86.4%), 108 (86.4%) and 109 (87.2%) patients on pre-contrast images alone (readers 1, 2 and 3, respectively) and in 117 (93.6%), 119 (95.2%) and 112 (89.6%) patients on combined pre- and post-contrast images, respectively. Highly significant (*P*<0.0001; all evaluations, all readers) improvements in lesion border delineation, visualization of lesion internal morphology and lesion conspicuity versus background were noted for assessments of combined pre- and post-contrast images compared to pre-contrast images alone (Table 3).

**Table 3:** Comparison of lesion visualization on pre- + post-contrast images versus pre-contrast images alone

Image quality		Reader 1 (*n*=107)	Reader 2 (*n*=107)	Reader 3 (*n*=106)
Lesion border delineation	Pre-contrast	2.9±0.80	2.5±0.78	2.9±0.67
	Pre- + post-contrast	3.7±0.45	3.3±0.55	3.8±0.38
	Change	0.8±0.78	0.8±0.84	0.9±0.67
	*P*-value	<0.0001	<0.0001	<0.0001
	95% CI of change	0.7–1.0	0.7–1.0	0.8–1.0
Visualization of lesion internal morphology	Pre-contrast	2.8±0.76	2.2±0.58	2.9±0.68
	Pre- + post-contrast	3.7±0.56	3.1±0.50	3.9±0.28
	Change	0.8±0.84	0.9±0.77	1.0±0.75
	*P*-value	<0.0001	<0.0001	<0.0001
	95% CI of change	0.7–1.0	0.7–1.0	0.9–1.2
Lesion conspicuity versus background	Pre-contrast	2.9±0.77	2.3±0.66	2.9±0.66
	Pre- + post-contrast	3.9±0.34	3.2±0.51	3.8±0.45
	Change	1.0±0.84	0.9±0.79	0.9±0.73
	*P*-value	<0.0001	<0.0001	<0.0001
	95% CI of change	0.8–1.1	0.8–1.1	0.8–1.1

Based on numbers of patients with available pre- and post-contrast scores. Values are mean±standard deviation of scores for each patient determined using 4-point scales from 1 to 4. *P*-value based on paired *t*-test for change from pre-contrast to pre- + post-contrast. *CI* confidence interval

Final diagnosis was available for 117 patients and included neoplastic disease (*n*=41), inflammatory or infectious disease (*n*=33), vascular disease (*n*=31), phakomatoses such as tuberous sclerosis (*n*=5), Sturge-Weber syndrome (*n*=2) and other (*n*=5). Readers 1, 2 and 3 accurately differentiated neoplastic from non-neoplastic disease in significantly more patients based on information available on combined pre- and post-contrast images than on information available on pre-contrast images alone (reader 1: 99/117 [84.6%] vs. 85/117 [72.7%], *P*=0.006; reader 2: 99/117 [84.6%] vs. 83/117 [70.9%], *P*=0.002; reader 3: 103/117 [88.0%] vs. 90/117 [76.9%], *P*=0.002). Similar results were obtained when each reader assigned a specific diagnosis to the imaging findings. Significantly more correct diagnoses were made based on information available on combined pre- and post-contrast images than on information available on pre-contrast images alone (reader 1: 93/117 [79.5%] vs. 78/117 [66.7%], *P*=0.01; reader 2: 79/117 [67.5%] vs. 55/117 [47.0%], *P*<0.0001; reader 3: 90/117 [76.9%] vs. 65/117 [55.6%], *P*<0.0001). Whereas none of the readers provided an accurate specific diagnosis for 25/117 (21.4%) patients based on evaluation of pre-contrast images alone, this number was significantly lower (12/117 [10.3%], *P*=0.02) when assessment was made of combined pre- and post-contrast images.

Full three-reader agreement for the correct differentiation of neoplastic from non-neoplastic disease was achieved for significantly (*P*=0.0001) more patients when assessment was based on combined pre- and post-contrast images (86/117 [73.5%]) than when assessment was based on unenhanced pre-contrast images alone (68/117 [58.1%]). Inter-reader agreement was good for assessments of both pre-contrast images alone (κ=0.612; 95% CI: 0.513, 0.711) and combined pre- and post-contrast images (κ=0.630; 95% CI: 0.529, 0.731). Similarly, three-reader agreement for correct specific lesion diagnosis was achieved for significantly (*P*<0.0001) more patients when assessment was based on combined pre- and post-contrast images (63/117 [53.8%]) than when assessment was based on unenhanced pre-contrast images alone (38/117 [32.5%]). Inter-reader agreement was fair for both assessments (κ=0.374, 95% CI: 0.252, 0.496 for assessment of pre-contrast images alone and κ=0.368, 95% CI: 0.220, 0.515 for assessment of combined pre- and post-contrast images).

**Fig. 1:**
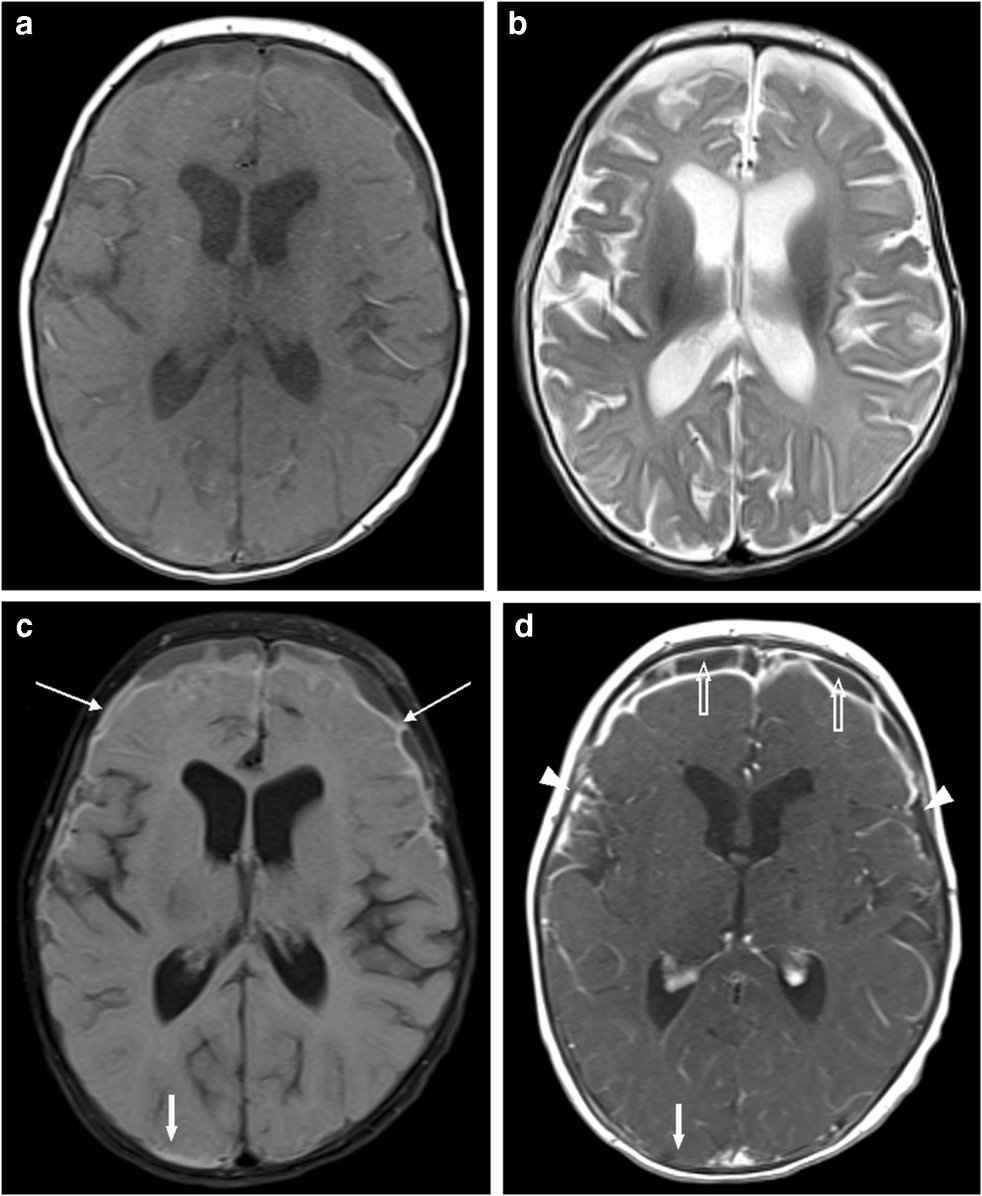
A 2-month-old girl with subdural empyema and meningitis. **a–c** Small subdural fluid collections in the frontal convexities bilaterally appear hypointense and hyperintense on the axial T1 (**a**) and T2 (**b**) images, respectively, with some areas of non-suppression on the axial fluid-attenuated inversion recovery (FLAIR) image (*thin arrows* in **c**) that suggest dural thickening and mild scalloping of the frontal cortical surface. FLAIR hyperintensity also extends slightly into the sulci suggestive of concomitant leptomeningeal disease. **d** The post-contrast axial T1 image shows subdural fluid collections (*open arrows*), and pachymeningeal and leptomeningeal enhancement (*arrowheads*). Note, a tiny sliver of FLAIR hyperintensity in the right occipital convexity (*thick arrow* in **c**) is not conspicuous on the post-contrast image (*solid arrow* in **d**), possibly reflecting a tiny fluid collection. Pachymeningeal and leptomeningeal enhancement seen on the post-contrast T1 image strongly suggest an infectious process and led to the imaging diagnosis of empyema and meningitis, which was later confirmed by cerebrospinal fluid analysis

**Fig. 2:**
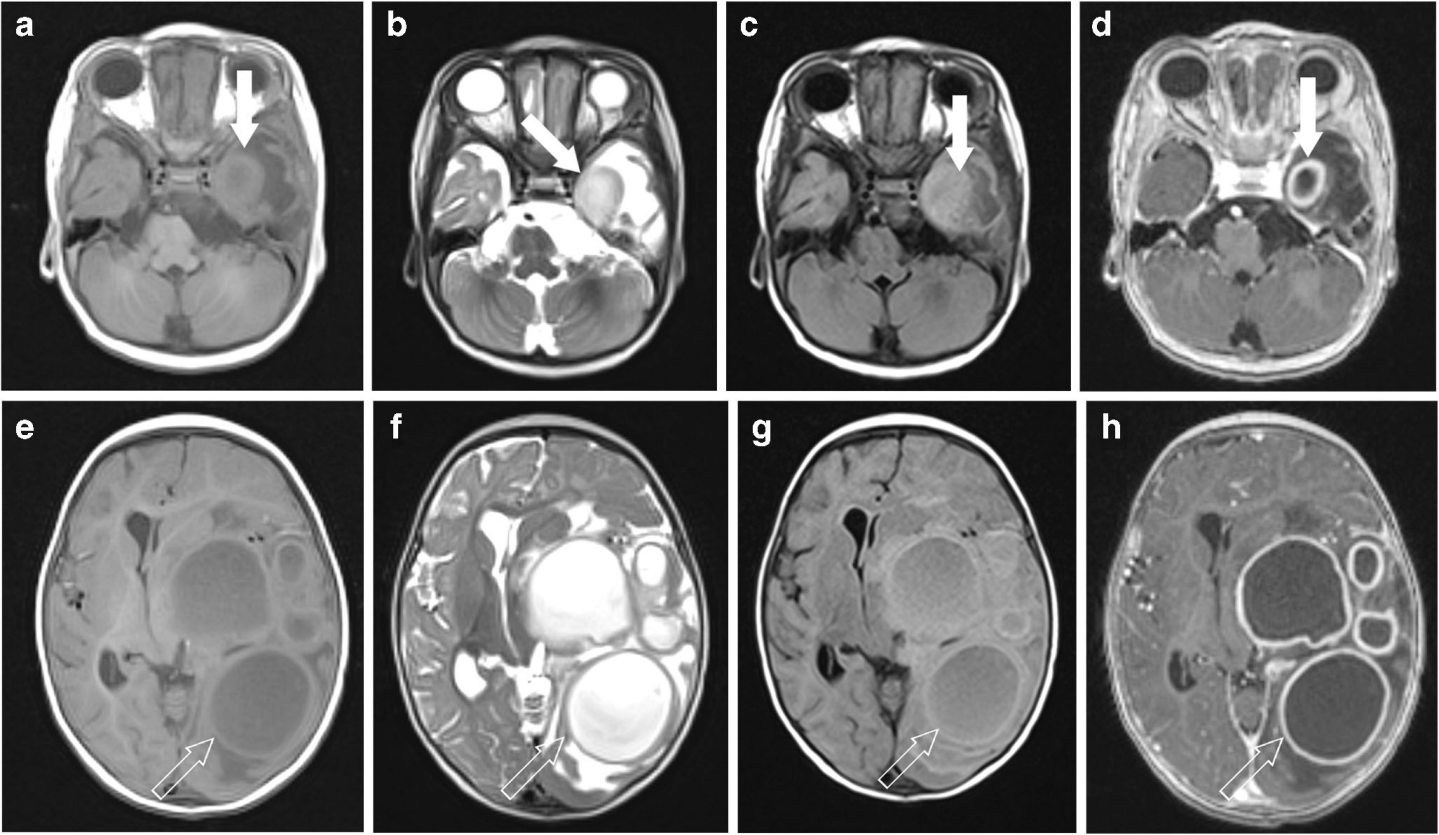
A 7-month-old boy with multifocal brain abscesses. **a–d** The lesion in the left temporal lobe (*solid arrows*) shows heterogeneous signal on the axial T1 (**a**) and T2 (**b**) images with mild hyperintensity and marked surrounding vasogenic edema on the axial fluid-attenuated inversion recovery (FLAIR) image (**c**). The post-contrast axial T1 image (**d**) clearly delineates a multifocal ring-enhancing lesion with thick but smooth peripheral enhancement characteristic of a cerebral abscess (likely pyogenic) in the left temporal lobe. **e–h** Multilocular, more cystic-appearing lesions are seen in the left frontal and parietal lobes (*open arrows*) with predominant T1 hypointensity (**e**) and T2 hyperintensity (**f**) but incomplete FLAIR signal suppression (**g**), associated with surrounding vasogenic edema, mass effect effacing the left lateral ventricle and rightward midline shift with mild trapping of the right lateral ventricle. The post-contrast T1 image (**h**) delineates multifocal ring-enhancing abscesses (likely pyogenic) in the left frontal and left parietal lobes. These findings are better delineated on post-contrast T1 images (**d** and **h**) than on pre-contrast T1 images (**a** and **e**)

**Fig. 3:**
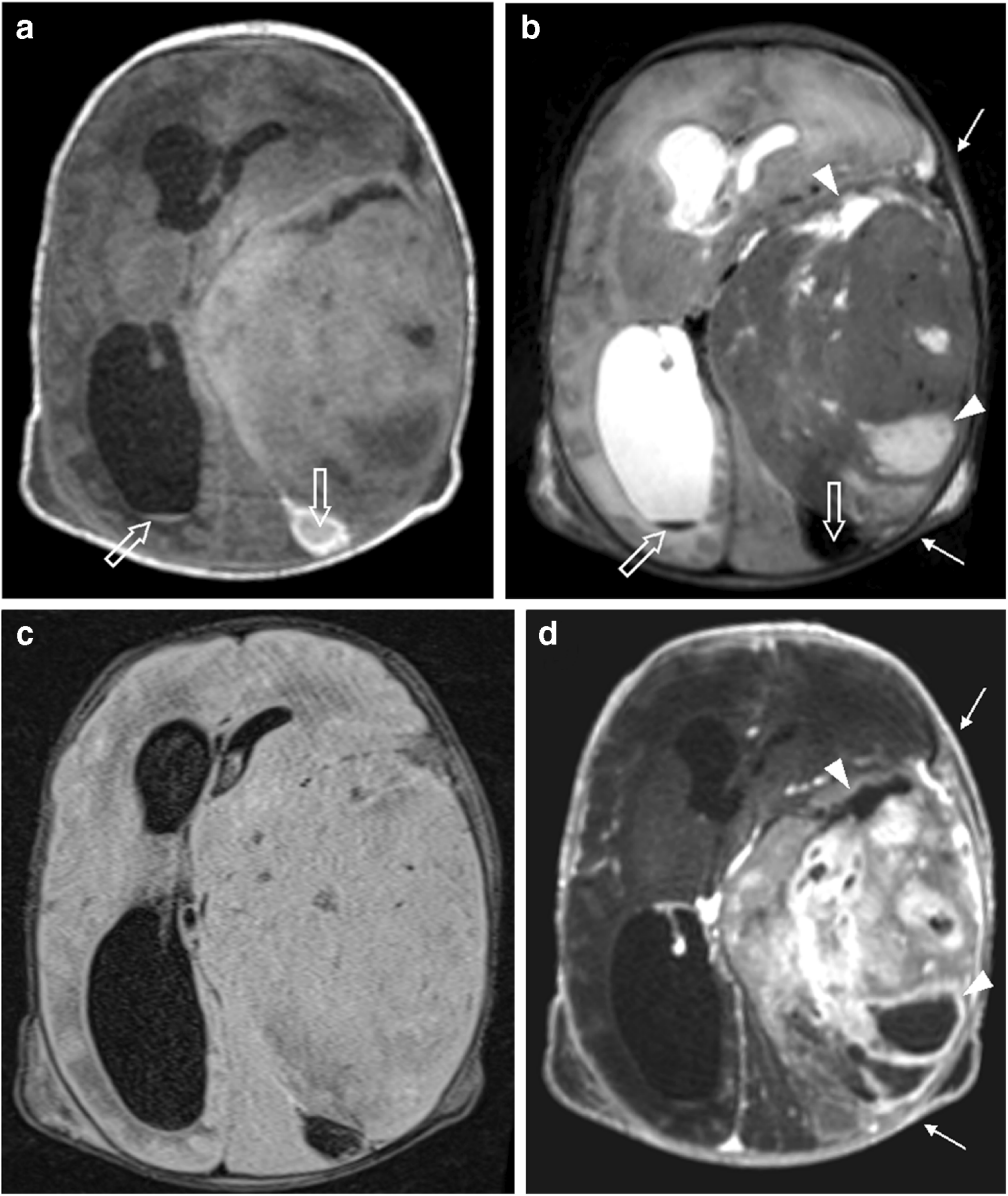
A 1-day-old girl with hypoxic-ischemic encephalopathy and a large left temporoparietal mass confirmed by surgery to be desmoplastic infantile ganglioglioma. **a–d** The mass is well circumscribed, peripherally (pial) based and mostly solid with heterogeneous signal showing iso- to mild hyperintensity on the pre-contrast axial T1 image (**a**) and predominant hypointensity (*solid arrows*) on the axial T2 image (**b**). Note a small amount of T1 hyperintense and T2 dark blood layering within the occipital horns (*open arrows*). Heterogeneously enhancing mass (*arrows*) and multiple internal non-enhancing cystic components (*arrowheads*) are poorly seen on the axial fluid-attenuated inversion recovery (FLAIR) image (**c**) but are depicted on T2 and clearly seen on the post-contrast axial T1 image (**d**). There is moderate mass effect and a rightward shift of midline structures causing near effacement of the left lateral ventricle and trapping of the right lateral ventricle but not significant surrounding edema. The mass and its extent are better delineated on the post-contrast T1 image (**d**) than on the pre-contrast T1 image (**a**)

Additional diagnostic information on images enhanced with gadoteridol was noted by readers 1, 2 and 3 for 118/125 (94.4%), 117/125 (93.6%) and 122/125 (97.6%) patients, respectively (Table 4). The primary benefits were in revealing abnormalities not seen on pre-contrast images and/or in improving lesion conspicuity (i.e. improving visualization of the size, extent and/or margins of a lesion) or better predicting the grade, histological type, vascularity and/or aggressiveness of a lesion (Figs. 1, 2 and 3).

**Table 4:** Additional information provided with the use of gadoteridol

	**Reader 1** ***n*** **(%)**	**Reader 2** ***n*** **(%)**	**Reader 3** ***n*** **(%)**
**Total number of patients assessed**^**a**^	125	125	125
Additional information: no	7 (5.6)	8 (6.4)	3 (2.4)
Additional information: yes	118 (94.4)	117 (93.6)	122 (97.6)
Patients with enhancing lesions	102 (81.6)	106 (84.8)	102 (81.6)
Patients with non-enhancing lesions	13 (10.4)	6 (4.8)	14 (11.2)
Patients with both enhancing lesions and non-enhancing lesions	2 (1.6)	1 (0.8)	7 (5.6)
Gadoteridol was helpful in excluding lesions (in patients with no lesion detected in both pre- and post-contrast images)	5 (4.0)	6 (4.8)	13 (10.4)
**Number of patients assessed with enhancement of lesions**^**a**^	102	106	102
Enhancement revealed an abnormality not seen on the pre-contrast MR images	30 (29.4)	32 (30.2)	28 (27.5)
Enhancement provided improved visualization of the size, extent and/or margins of a lesion (better conspicuity)	49 (48.0)	81 (76.4)	98 (96.1)
The pattern of enhancement was useful in predicting the grade, histological type, vascularity and/or aggressiveness of a lesion	43 (42.2)	65 (61.3)	75 (73.5)
Enhancement suggested residual tumor in an operative site not distinguishable from postsurgical changes on pre-contrast images	2 (2.0)	0	0
Enhancement documented the activity or aggressiveness of certain non-neoplastic processes, including multiple sclerosis, vasculitis and infection	16 (15.7)	1 (0.9)	17 (16.7)
Enhancement proved the subacute nature of a lacuna or infarct when the age of such lesion was clinically and radiologically indeterminate	6 (5.9)	3 (2.8)	5 (4.9)
Other diagnostic benefits of enhancement	21 (20.6)	0	0
**Number of patients assessed with no enhancement of lesions**^**a**^	13	6	14
Lack of enhancement indicated a benign or low-grade nature of the mass rather than high-grade nature	2 (15.4)	4 (66.7)	8 (57.1)
Lack of enhancement provided support that high-grade tumor had been completely resected from an operative site	1 (7.7)	1 (16.7)	0
Lack of enhancement clarified the relative inactivity or low aggressiveness of white matter lesions in a clinical setting in which their benign nature could be presumed (documents systemic malignancy, vasculitis or demyelinating disease)	3 (23.1)	0	1 (7.1)
Lack of enhancement proved the remote nature of a lacuna or infarct when the age of such lesion was otherwise radiologically indeterminate but questioned clinically	0	0	3 (21.4)
In the setting of a possible central nervous system infection, lack of enhancement helped by excluding extensive meningeal involvement, pseudomembrane formation or abscesses	0	0	4 (28.6)
Other diagnostic benefits of lack of enhancement in the appropriate clinical setting	8 (61.5)	1 (16.7)	2 (14.3)

A subject may have findings in more than one category

^a^Denominator for percentages

### Safety findings

Six (4.8%) of 125 children who received gadoteridol experienced 11 adverse events during the prospectively defined 48-h monitoring period as adjudicated by the investigating radiologist. All adverse events were changes in laboratory values. One further child experienced two changes in laboratory values at 66 h after gadoteridol administration that were also considered adverse events. Although these events occurred outside the 48-h monitoring period, they were nevertheless included in the final analysis, giving an overall incidence of 5.6% (7/125) and 13 adverse events. Each of these adverse events was categorized by the investigating radiologist as mild, apart from one adverse event that was categorized as moderate and one adverse event for which categorization was not available.

In five of these seven patients, the investigating radiologist considered there to be “no reasonable possibility” of a relationship between the reported adverse event (nine adverse events overall) and the administration of gadoteridol. The laboratory changes in these five patients included one patient with elevated platelet levels of unreported intensity at 40 h after the MRI examination that returned to normal levels within 24 h; one patient with a mild decrease in hemoglobin at 29 h that returned to normal levels; one patient with mild decreases in hemoglobin and hematocrit at 17 h that were ascribed to administered antibiotics and that returned to normal levels; one patient with mild decreases in hemoglobin, hematocrit and red blood cell count at 33 h that were ascribed to blood loss during surgery that was recovered by transfusion; and one patient with moderately lowered platelet levels and mildly elevated blood chloride at 66 h that were ascribed to blood loss during surgery that was recovered by transfusion.

In two patients (1.6%), the relationship of the adverse event to gadoteridol administration could not be excluded, although in both cases the investigating radiologist considered there to be doubtful or insufficient/poor evidence of a causal relationship. One of these cases was a 5-month-old infant with lowered hemoglobin at 4 h after gadoteridol administration and mildly elevated blood chloride and blood sodium at 12 h after the MRI examination, which were considered unrelated to gadoteridol administration. The other case was an 11-month-old infant with normal serum creatinine values and lowered urea nitrogen 16 h after gadoteridol administration. Both events were mild. No clinically meaningful changes were recorded by the investigators for any patient with vital sign or ECG data available. There were no reports of sedation- or anesthesia-related adverse events and all patients recovered without sequelae.

## Discussion

Neonates and young infants represent an extremely vulnerable population for contrast-enhanced imaging procedures in part because of their size and fragility, and in part because the hepatic and renal clearance mechanisms necessary for the elimination of contrast materials are relatively underdeveloped [22, 23]. The macrocyclic GBCAs widely used for contrast-enhanced MRI in pediatric subjects are eliminated exclusively via the kidneys. Although pharmacokinetic studies of renally excreted drugs, including GBCAs, in healthy newborn infants reveal kinetic behavior comparable to that in older children and adults [24–26], the fact that eGFR roughly equivalent to adult levels does not occur until about 6 months [23] means that attention appropriately centers not only on the acute safety and efficacy of GBCAs in this population but also on the potential longer-term effects of GBCA administration. Our study of 125 children aged 2 years and younger, including 57 children younger than 6 months, revealed only 13 changes in laboratory values in 7 children that were classified as adverse events. Of these 13 events, 11 were considered unrelated to the administration of gadoteridol. Only two altered laboratory values in two patients were reported as possibly related adverse events, but these were considered doubtful by the investigating radiologist. There was no impact on serum creatinine levels and no effects on vital signs or ECG recordings in those infants in whom determinations were made. In terms of safety, our findings confirm the low incidence of acute/subacute adverse events reported by Cho et al. [11] in adult and pediatric subjects undergoing routine contrast-enhanced MRI with gadoteridol. Moreover, they bear excellent comparison with findings for other macrocyclic GBCAs in children under 2 years of age [17, 25–28].

Our results confirm that gadoteridol is effective not only for differentiating neoplastic from non-neoplastic disease (83.8–86.3% accuracy for assessing combined pre- plus post-contrast images; all readers) but also for the more challenging assessment of accuracy for specific diagnosis (68.4–80.3% accuracy on combined pre- plus post-contrast images compared to only 47.0–67.5% accuracy for assessment of pre-contrast images alone). Of note, the images in this study were assessed in a fully independent manner with the readers blinded to the medical history and clinical characteristics of the patients, creating a greater diagnostic challenge in an artificial manner compared to the usual clinical setting. Future work might look at whether, and to what extent, diagnostic performance is improved on pre- plus post-contrast images relative to pre-contrast images alone if readers are fully aware of the medical history and clinical characteristics of the patients.

Unfortunately, the lack of similar diagnostic efficacy studies with other macrocyclic GBCAs in a similar pediatric population precludes direct comparison of our findings with other macrocyclic GBCAs. However, it is worth noting that the improvement in lesion visualization seen on combined pre- plus post-contrast images relative to pre-contrast images alone bears excellent comparison with subjective efficacy results reported for the macrocyclic GBCAs gadoterate meglumine [25] and gadobutrol [26] in children aged 2 years and younger. Furthermore, the same lesion visualization parameters evaluated in this study (lesion border delineation, visualization of lesion internal morphology and lesion conspicuity versus background) were also evaluated in a previous large-scale multi-center intra-individual crossover comparison of 0.1 mmol/kg gadoteridol and 0.1 mmol/kg gadobutrol (Gadavist/Gadovist; Bayer) in adults referred for contrast-enhanced MRI of the CNS [10]. The results of that study revealed no significant differences between these two GBCAs either for lesion visualization or diagnostic performance [10]. The similar lesion visualization performances of gadoteridol and gadobutrol are recognized in the current prescribing information for gadobutrol as reviewed by the FDA, which states, “Performances of Gadavist and gadoteridol for visualization parameters were similar” [29].

Given that the safety and efficacy profiles of the three macrocyclic GBCAs are essentially equivalent and assuming no relevant differences in non-radiologic factors (e.g., availability, price), the choice of which GBCA to use in neonates and young children might conceivably come down to concern over the potential long-term risks associated with gadolinium retention. Although studies in adults receiving multiple GBCA doses do not reveal any evidence of harm associated with gadolinium retention [1–4], no data are available on the potential long-term impact of GBCA administration on very young pediatric subjects. As noted by others, possible long-term effects of retained gadolinium are potentially of greater concern in this population given their increased vulnerability from continuing development, longer life expectancy and potentially longer period of exposure [30, 31]. Although studies in animals have shown that the levels of retained gadolinium are much lower after administration of macrocyclic GBCAs than after administration of certain linear GBCAs [12, 13, 32, 33], a recent evaluation of autopsy samples from children who received between 1 and 20 GBCA administrations has shown that gadolinium is retained in the brains of children even after the administration of just a single dose of macrocyclic GBCA [30]. In the absence of any data concerning the clinical significance of this retention, when a contrast-enhanced MRI examination is deemed necessary it would seem prudent to select the GBCA that is cleared rapidly from the brain and body leading to lower levels of retained gadolinium during the first months and years after administration. Of the three macrocyclic GBCAs available for clinical use, gadoteridol has been shown in numerous animal studies to clear most rapidly from brain and other body tissues [12–15]. Although reasons for the more rapid clearance remain unclear, it is possible the specific molecular features of the gadoteridol molecule (low molecular weight and viscosity, neutrality and high lipophilicity) are sufficient to promote more rapid elimination from the brain and other soft body tissues [14, 15, 34, 35] and that this results in lower levels of retained gadolinium in the first weeks/months after exposure. Clearly, a lower amount of gadolinium retained in rat brain and body tissues in the first weeks/months after gadoteridol administration would equate to several years in humans, given that one rat year equates to roughly 30 human years [36] if findings in animals are considered indicative of the human situation. This may be relevant if future studies do indeed demonstrate a negative impact of GBCA exposure on human health.

A limitation of our study is that patients were enrolled retrospectively. However, a patient cohort free of selection bias was ensured by the approach to patient inclusion, with patients enrolled consecutively in strict reverse chronological order from the date of local IRB approval until the prospectively defined target enrollment was attained. Thereafter, image assessment was performed prospectively by three independent readers who were unaffiliated with the enrollment centers and fully blinded to all patient information. A second limitation is that our study was insufficiently powered to assess safety in terms of allergic-like reactions. Although a study by Cho et al. [11] in 6,163 patients, including 52 pediatric patients between 2 and 18 years of age, suggests that the incidence of allergic-like reactions to gadoteridol is comparable to incidences observed after exposure to other macrocyclic GBCAs, to our knowledge no adequately powered prospective studies have been performed to investigate the incidence of allergic-like reactions to any GBCA in children younger than 2 years of age. Third, we did not include diffusion tensor imaging (DTI) or diffusion-weighted imaging (DWI) in the evaluation. DTI/DWI sequences are increasingly used in clinical routine and may have provided additional information to further aid lesion diagnosis and characterization. Finally, we did not look for signs of T1 hyperintensity in the dentate nucleus or globus pallidus or for any potential long-term effects of gadoteridol exposure. In part, this reflects the fact that brain T1 hyperintensity after exposure to macrocyclic GBCAs has been reported relatively infrequently in pediatric subjects and, to our knowledge, never after exposure to gadoteridol [37]. Moreover, when T1 hyperintensity has been reported after exposure to macrocyclic GBCAs, it has invariably been after multiple (typically >4) exposures [38, 39]. Almost all children enrolled in our study underwent only one gadoteridol-enhanced MRI examination. Among those children who underwent two or more examinations, only the first examination that showed an enhancing lesion was evaluated. Assessment of the potential long-term effects of exposure to gadoteridol was beyond the scope of the study but should be addressed in future longitudinal investigations.

## Conclusion

Our study confirms that gadoteridol administered at a dose of 0.1 mmol/kg body weight is safe, well tolerated and effective in patients ≤2 years of age.
